# Daily egg consumption in hyperlipidemic adults - Effects on endothelial function and cardiovascular risk

**DOI:** 10.1186/1475-2891-9-28

**Published:** 2010-07-02

**Authors:** Valentine Njike, Zubaida Faridi, Suparna Dutta, Anjelica L Gonzalez-Simon, David L Katz

**Affiliations:** 1 Yale-Griffin Prevention Research Center, 130 Division Street, Derby, CT 06418, USA; 2 Department of Epidemiology and Public Health, Yale University School of Medicine, 60 College St, New Haven, CT 06510-3210, USA; 3 Griffin Hospital, 130 Division Street, Derby, CT 06418, USA; 4 Department of Biomedical Engineering, Yale University, 55 Prospect St, New Haven, CT 06511-6816, USA

## Abstract

**Background:**

Limiting consumption of eggs, which are high in cholesterol, is generally recommended to reduce risk of cardiovascular disease. However, recent evidence suggests that dietary cholesterol has limited influence on serum cholesterol or cardiac risk.

**Objective:**

To assess the effects of egg consumption on endothelial function and serum lipids in hyperlipidemic adults.

**Methods:**

Randomized, placebo-controlled crossover trial of 40 hyperlipidemic adults (24 women, 16 men; average age = 59.9 ± 9.6 years; weight = 76.3 ± 21.8 kilograms; total cholesterol = 244 ± 24 mg/dL). In the acute phase, participants were randomly assigned to one of the two sequences of a single dose of three medium hardboiled eggs and a sausage/cheese breakfast sandwich. In the sustained phase, participants were then randomly assigned to one of the two sequences of two medium hardboiled eggs and 1/2 cup of egg substitute daily for six weeks. Each treatment assignment was separated by a four-week washout period. Outcome measures of interest were endothelial function measured as flow mediated dilatation (FMD) and lipid panel.

**Results:**

Single dose egg consumption had no effects on endothelial function as compared to sausage/cheese (0.4 ± 1.9 vs. 0.4 ± 2.4%; *p *= 0.99). Daily consumption of egg substitute for 6 weeks significantly improved endothelial function as compared to egg (1.0 ± 1.2% vs. -0.1 ± 1.5%; *p *< 0.01) and lowered serum total cholesterol (-18 ± 18 vs. -5 ± 21 mg/dL; *p *< 0.01) and LDL (-14 ± 20 vs. -2 ± 19 mg/dL; *p *= 0.01). Study results (positive or negative) are expressed in terms of change relative to baseline.

**Conclusions:**

Egg consumption was found to be non-detrimental to endothelial function and serum lipids in hyperlipidemic adults, while egg substitute consumption was beneficial.

## Background

As of the early 1970's, a reduction in consumption of eggs, a concentrated source of cholesterol (one yolk provides ~215 mg of cholesterol), had been widely recommended in an effort to lower blood cholesterol and reduce the risk of heart disease[1]. In 1973, the American Heart Association (AHA) guidelines specifically advocated exclusion of eggs from the diet, accompanying the advised cholesterol restriction[2]. More recent AHA guidelines no longer advise for or against egg or egg yolk consumption, admitting that there is a lack of scientific evidence for selecting a target level for dietary cholesterol[3]. This is partially due to individual differences in serum cholesterol responses to dietary cholesterol. The recommended intake of daily dietary cholesterol continues to be 300 mg/day or less for healthy adults and less than 200 mg/day for persons with elevated cholesterol or heart disease[3]. Given the widespread nature of this recommendation, there is surprisingly little evidence that egg consumption increases blood cholesterol levels, thereby increasing cardiovascular risk [4].

Data from recent studies show that consumption of one or two eggs per day, when part of a low fat diet, does not adversely affect the lipid profile[5,6]. In fact, the preclusion of eggs from the diet may represent a potential reduction in overall dietary quality. As an inexpensive functional food with an exceptional nutritional profile[7,8], eggs are an excellent natural source of folate, riboflavin, selenium, choline, vitamin B12, and fat-soluble vitamins A, D, E, and K. Eggs also provide high-quality, bioavailable protein[9,10] with little total fat. Compared to other animal protein sources, eggs contain proportionately less saturated fat, which has generally been recognized as a strong dietary determinant of elevated low-density lipoprotein (LDL) levels and increased risk of coronary heart disease (CHD) [11] although this topic is not without controversy [12].

As a dietary substitute for eggs, egg substitute is comprised of 99% egg whites and provide 12 key vitamins and nutrients, including riboflavin, B12, folate, and pantothenic acid, while excluding the cholesterol contribution of the egg yolk[13]. Although nutritionally similar to eggs, egg substitute contains emulsifiers, stabilizers, and artificial color and are on average three times as expensive as regular eggs.

Endothelial function refers to arterial vasomotor responses mediated predominantly by the release of nitric oxide (vasodilating), and endothelin (vasoconstricting) from the vascular endothelium[14,15], and plays an important role in the pathogenesis of atherosclerosis, hypertension, cardiovascular disease, and diabetes[15-17]. Endothelial dysfunction correlates strongly with both coronary disease and its risk factors[18,19] and reverses in response to risk modification efforts[15,20-27]. Endothelial dysfunction has increasingly been viewed as an indicator of coronary risk[19], and its amelioration as an indicator of risk reduction[20,23].

The relationship of egg consumption to coronary outcomes depends not only on the cholesterol content of eggs themselves, but on the composition of the total diet. It is a common misconception that dietary cholesterol increases serum cholesterol which increases CHD risk[28,29]; however, research has failed to provide substantial evidence of this assumed relationship[30]. In our previous trial, daily ingestion of eggs did not produce adverse effects on cardiac risk, as indicated by endothelial function and lipid profile, in healthy adults[31]. To the best of our knowledge, no study has ever compared the effects of egg versus egg substitute consumption on cardiovascular risk. Therefore, we performed a randomized cross-over trial to assess the effects of egg or egg substitute consumption on endothelial function and lipid panel in hyperlipidemic adults.

## Subjects and Methods

### Subjects

Forty adults (16 men and 24 women) with diagnosed hyperlipidemia were recruited from Southwestern Connecticut; largely through mass media print advertisements and posters. Eligible subjects were 35 years of age or older if they were male or post-menopausal and not currently using hormone replacement therapy if they were female. Additionally, eligible subjects were non-smokers, and hyperlipidemic as defined by serum total cholesterol >240 mg/dL, and/or LDL cholesterol >160 mg/dL, and/or a total cholesterol/HDL ratio >5.7. All ethnic and minority groups were equally eligible. Individuals with a current eating disorder, a restricted diet, diagnosed coronary disease, diabetes, or sleep apnea were excluded from the study. Additional exclusion criteria included the regular use of lipid-lowering medication, insulin or glucose sensitizing medication, vasoactive medication or nutriceuticals, high dose vitamin E or C, and fiber supplements.

**Figure 1:**
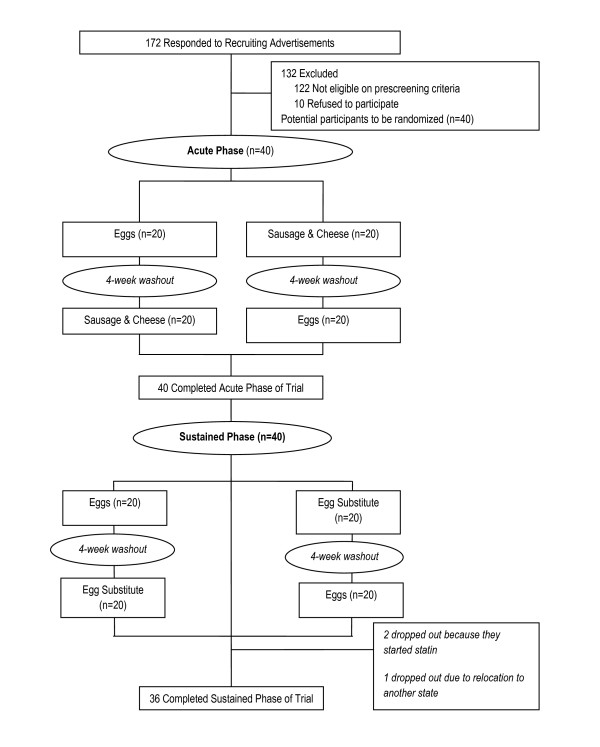
**Flow of Participants through the Trial**.

Individuals who responded to recruitment efforts (n = 172) were pre-screened using a semi-structured telephone interview. Those who met initial screening criteria (n = 40) underwent a clinical screening evaluation (weight, height, body mass index (BMI), and blood pressure measurements) performed by a clinical research specialist, along with laboratory testing (fasting total cholesterol, HDL, LDL, and triglycerides levels) (Figure 1).

All participants provided informed consent and were compensated monetarily for their time. The study protocol was approved by the Institutional Review Board (IRB) of Griffin Hospital (Derby, CT).

### Study Design

This study was a randomized, single-blind crossover trial with investigators blinded to treatment assignments. The trial consisted of an acute and a sustained phase. In the acute phase, 40 participants were randomly assigned to consume one of the two sequence permutations of a single dose of breakfast of three medium hardboiled eggs and a sausage/cheese breakfast sandwich (Table 1). In the sustained phase, participants were randomly assigned to one of the two sequence permutations of two medium hardboiled eggs and 1/2 cup of egg substitute breakfast daily for six weeks. Randomization was conducted by the data manager using a SAS (SAS version 9.1; SAS Institute, Cary, NC) algorithm. Each treatment assignment was separated by a four-week washout period. The study participants fasted overnight before undergoing endothelial function assessment. Due to the obvious dietary makeup of each treatment assignment, it was not possible to blind participants to their assignment; however, the ultrasonographer was strictly blinded to participants' treatment assignment.

**Table 1:** Composition of the Breakfasts in the Acute Phase

Nutrition Content	Sausage & Cheese	3 Medium Eggs
Calories (kcal)	310	189

Calories from fat (Kcal)	260	118

Fat (gm)	29	13

Saturated fat (gm)	12	4

Sodium (mg)	720	185

Protein (g)	13	17

Calories from protein (kcal)	52	66

Carbohydrate (g)	0	1

### Outcome Measures

#### Endothelial Function Assessment

The brachial artery reactivity studies (BARS) methodology employed is comparable to those of other leading labs[16,32-36], and is described in "Guidelines for ultrasound assessment of endothelial-dependent flow-mediated vasodilation of the brachial artery"[16]. Participants were required to lie at rest in the quiet, temperature-controlled, softly lit room for at least 15 minutes before scanning was initiated. The baseline diameter of the brachial artery was measured from two-dimensional ultrasound images using a high frequency, 10-15 MHz, vascular ultrasound transducer (Sonos 4500; Phillips Medical Systems, Andover, MA). Arterial flow-velocity was measured by means of a pulsed Doppler signal at a 70° angle to the vessel, with the range gate in the center of the artery. Flow was determined by multiplying the arterial cross-sectional area (πr^2^) by the Doppler flow velocity. The timing of each image frame with respect to the cardiac cycle was determined with simultaneous ECG gating during image acquisition via the high-quality mainframe ultrasound system. The arterial diameter was measured at a fixed distance from an anatomical marker, such as a bifurcation, with ultrasonic calipers. Measurements were taken from the anterior to the posterior "m" line in diastole. The brachial artery was imaged at a location 3-7 cm above the antecubital fossa in the longitudinal plane. A segment with clear anterior and posterior intimal interfaces between the lumen and vessel wall was selected for continuous 2D gray scale imaging. The transmit (focus) zone was set to the depth of the near wall because of difficulty in differentiating the near from the far wall "m" line (the interface between media and adventitia)[16,33]. Images were acquired on videotape and magnetic optical disk for evaluation and analysis subsequent to the examination. Diameter was obtained from m-line to m-line, over a consistent segment of vessel at least 10-15 mm in length.

To create a flow stimulus in the brachial artery, a sphygmomanometer cuff was placed on the upper arm proximal to the transducer. A baseline blood flow and diameter were acquired. Arterial occlusion was created by cuff inflation to 50 mm Hg above the systolic blood pressure. The cuff remains inflated for 5 minutes. This causes ischemia and consequent dilation of downstream resistance vessels via auto-regulatory mechanisms[16]. Cuff deflation induces a brief high-flow state through the brachial artery (reactive hyperemia) to accommodate the dilated resistance vessels. The resulting increase in shear stress causes the normal brachial artery to dilate. A pulsed Doppler signal was obtained within 15 seconds of cuff release to assess hyperemic velocity, and a longitudinal image of the artery was recorded continuously from 20 seconds to 2 minutes after cuff deflation. All images were coordinated with a continuous ECG monitor and obtained at end-diastole. The resultant coefficient of intra observer reliability was 0.9.

Flow-mediated dilation (FMD) was measured as the percent change in brachial artery diameter from pre-cuff inflation to 60-seconds post-cuff release. In addition to brachial diameter at 60 seconds post-cuff release, flow after cuff deflation within the first 15 seconds was used as an indicator of stimulus strength, hyperemic flow being the stimulus for endothelial reactivity. To account for potential variability in stimulus strength, FMD was divided by flow at 15 seconds[16]. post-cuff deflation to create a stimulus-adjusted response measure[31,37,38].

##### Lipid Profile

Serum was drawn for lipid assessments about twenty minutes prior to endothelial function assessment. The lipid profile was determined as follows: Total cholesterol (Tchol), triglycerides (TRIG), and high-density lipoprotein (HDL) were obtained by direct measurements. Very-low-density lipoprotein (VLDL) and low-density-lipoprotein (LDL) were obtained by calculation: VLDL = TRIG/5; and LDL = Tchol - (VLDL + HDL) [39].

##### Body Weight

Body weight was measured for all study participants at the beginning and end of the sustained phase. Body weight was measured to the nearest 0.5 pound using a balance-type medical scale. Participants were measured in the morning, unclothed with the exception of undergarments.

##### Blood Pressure

Blood pressure was determined with the use of the Datascope Accutorr Plus automatic digital blood pressure device (Datascope Corp, Mahwah, NJ) with the participant supine after a 5-min period of rest. Both systolic and diastolic pressures were calculated as the mean value of 2 readings 5 minutes apart. All measurements were obtained by one investigator.

### Statistical Analysis

Statistical analysis was conducted using SAS software (Version 9.1, SAS Institute, Cary, NC). A two-tailed *p*-value of ≤ 0.05 was considered statistically significant. Two-way repeated measures ANOVA, with treatment and time as the main effects, were performed to compare treatment-specific outcome measures responses, accounting for time differences. Within-treatment effects for outcome measures were assessed using paired *t*-tests. The combined effect of independent variables (age, blood pressure, LDL, BMI and treatment sequence) and treatment assignment on all outcome measures was assessed with generalized linear modeling. All analyses of endpoints were based on the intention-to-treat principle.

Sample size was predicated on 80% power to detect a minimal difference of 3.5% change in FMD between the egg and egg substitute treatments at six weeks. A two-tailed alpha level of 0.05 was set with an allowance for 10% attrition and noncompliance.

## Results

Forty hyperlipidemic participants participated in this study. Sixty percent of the participants were female. Participants ranged in age from 35 to 77 years, with a mean age of 60 years (Table 2). Four participants dropped out of the study after the acute phase. One participant dropped out because the participant was unwilling to consume eggs or egg substitute daily for six weeks, another dropped out because of relocation to another state, and two dropped out because they started using lipid lowering medication (statin).

**Table 2:** Demographic and Baseline Characteristics

Variable	Values	Range
Gender		
Female	24 (60%)	
Male	16 (40%)	
Race		
White	39 (97.5%)	
African American	1 (2.5%)	
Age (years)	59.9 ± 9.6	35 to 77
Brachial Artery Diameter (mm)	4.0 ± 0.8	2.8 to 5.4
Systolic Blood Pressure (mmHg)	131.4 ± 15.7	94 to 173
Diastolic Blood Pressure (mmHg)	73.0 ± 12.6	45 to 105
Framingham 10-years Risk (%)	6.6 ± 5.8	1 to 30
Body Mass Index (kg/m^2^)	28.7 ± 4.7	20.4 to 38.4
Weight (kg)	76.3 ± 21.8	45 to 105

Values are mean ± SD except otherwise stated

### Acute Phase

After a single dose of eggs, endothelial function did not change from baseline as compared to sausage and cheese ( *p *= 0.99). Accounting for the strength of the stimulus that determines vasodilatations (SARM), our findings on endothelial function persisted (Table 3.)

**Table 3:** Acute Phase: Mean change in Outcome Measures after Treatment Assignment

Variable	Egg (n = 40)	Sausage & Cheese (n = 40)	p-value
Baseline Brachial Artery Diameter (mm)	4.0 ± 0.8	4.0 ± 0.7	0.79*
Flow Mediated Dilatation (%)			
Baseline	5.9 ± 4.6	5.2 ± 3.6	0.45*
Post-prandial	6.3 ± 5.3	5.6 ± 4.5	
Change	0.4 ± 1.9 (*P *= 0.22)	0.4 ± 2.4 (*P *= 0.34)	0.99
Adjusted change†	0.3 ± 2.1 (*P *= 0.46)	0.4 ± 2.1 (*P *= 0.31)	0.84
Stimulus adjusted response measure			
Baseline	0.10 ± 0.12	0.06 ± 0.06	0.05*
Post-prandial	0.08 ± 0.09	0.07 ± 0.06	
Change	-0.02 ± 0.11 (*P *= 0.31)	0.00 ± 0.08 (*P *= 0.89)	0.35

Values are mean ± SD; p-value obtained from ANOVA for repeated measurements except otherwise stated; Change = Post-prandial - Baseline; *p-value obtain from Student's t-test; p-values in parenthesis indicate within-group p-values; † obtained from generalized linear models, controlling for age, blood pressure, LDL and BMI

### Sustained Phase

Daily consumption of egg substitute for six weeks improved endothelial function relative to egg consumption ( *p *< 0.01). These findings persisted controlling for the variation of the strength of the stimulus that causes the vasodilatation (Table 4.)

**Table 4:** Sustained Phase: Mean Change in Outcome Measures after Six Weeks of Treatment

Variable	Egg (n = 36)	Egg substitute (n = 36)	p-value
*Endothelial Function*			
Flow Mediated Dilatation (%)			
Baseline	5.6 ± 3.9	5.8 ± 3.9	0.78
6 Weeks	5.3 ± 4.1	6.9 ± 4.0	
Change	-0.1 ± 1.5 (*P *= 0.80)	1.0 ± 1.2 (*P *< 0.01)	<0.01
Adjusted change†	-0.2 ± 1.3 (*P *= 0.35)	0.9 ± 1.4 (*P *< 0.01)	< 0.01
Stimulus adjusted response measure			
Baseline	0.08 ± 0.10	0.06 ± 0.06	0.39
6 Weeks	0.08 ± 0.11	0.09 ± 0.09	
Change	0.01 ± 0.05 (*P *= 0.54)	0.03 ± 0.06 (*P *< 0.01)	0.07
*Lipid Panel*			
Total Cholesterol (mg/dL)			
Baseline	244 ± 24	244 ± 24	1.00
6 Weeks	239 ± 27	227 ± 27	
Change	-5 ± 21 (*P *= 0.10)	-18 ± 18 (*P *< 0.01)	< 0.01
Low Density Lipoprotein (mg/dL)			
Baseline	168 ± 17	168 ± 17	
6 Weeks	165 ± 24	154 ± 24	
Change	-2 ± 19 (*P *= 0.30)	-14 ± 20 (*P *< 0.01)	0.01
High Density Lipoprotein (mg/dL)			
Baseline	52 ± 15	52 ± 15	1.00
6 Weeks	51 ± 14	50 ± 13	
Change	-1 ± 11 (*P *= 0.53)	-2 ± 10 (*P *= 0.15)	0.63
Triglycerides (mg/dL)			
Baseline	132 ± 52	132 ± 52	1.00
6 Weeks	118 ± 47	116 ± 50	
Change	-14 ± 37 (*P *= 0.02)	-18 ± 43 (*P *= 0.03)	0.83
Total Cholesterol to High Density Lipoprotein Ratio			
Baseline	5.0 ± 1.3	5.0 ± 1.3	1.00
6 Weeks	5.0 ± 1.2	4.8 ± 1.3	
Change	-0.06 ± 0.66 (*P *= 0.54)	-0.21 ± 0.82 (*P *= 0.11)	0.38
*Body composition*			
Weight (kg)			
Baseline	81 ± 19	81 ± 19	1.00
6 Weeks	82 ± 18	82 ± 18	
Change	0.4 ± 2.3 (*P *= 0.33)	0.7 ± 2.4 (*P *= 0.08)	0.52
Body Mass Index (kg/m^2^)			
Baseline	29.2 ± 4.5	29.2 ± 4.5	1.00
6 Weeks	29.3 ± 4.3	29.5 ± 4.5	
Change	0.2 ± 0.8 (*P *= 0.13)	0.4 ± 0.9 (*P *= 0.04)	0.56

Values are mean ± SD; p-value obtained from ANOVA for repeated measurements except otherwise stated; p-values in parenthesis indicate within-group p-values; *p-value obtain from student ttest; Change = 6 Weeks - Baseline; † obtained from generalized linear models, controlling for age, blood pressure, LDL and BMI

Daily consumption of egg substitute for six weeks significantly lowered total cholesterol as compared to egg consumption ( *p *< 0.01) and also lowered LDL as compared to egg consumption ( *p *= 0.01). However, daily consumption of egg substitute for six weeks did not significantly lowered total cholesterol to HDL ratio as compared to egg consumption ( *p *= 0.38).

Daily consumption of egg or egg substitute for six weeks did not show significant increase in BMI as compared to egg consumption (*p *= 0.56)

## Discussion

Our findings in this study expand on existing evidence that short-term egg consumption does not adversely affect endothelial function, in a population not previously examined: hyperlipidemic adults. Moreover, we observed that consuming eggs daily did not unfavorably influence serum cholesterol or other measures of the lipid profile. While the subjects demonstrated impaired endothelial function at baseline (i.e. relative to healthy endothelial function), the acute induction of endothelial dysfunction by the test meal high in saturated fat was not observed. Egg substitute, which is made from 99% egg whites, is lower in calories relative to whole eggs, lacks cholesterol and fat, and is fortified with vitamins, also lowered cholesterol and triglyceride levels. In addition, egg substitute led to a decrease in LDL and significantly improved endothelial function, as compared to sustained egg consumption. To the best of our knowledge, this is the first study to provide evidence that egg or egg substitute consumption does not adversely affect endothelial function in hyperlipidemic adults.

The acute phase findings were consistent with those of the sustained phase. Single, acute doses of egg did not adversely affect endothelial function. The sausage and cheese breakfast sandwich, designed to demonstrate acute dysfunction in the endothelium, surprisingly also did not adversely affect endothelial function. This finding is consistent with the prior study of egg ingestion in healthy adults conducted at our lab[31], but is at odds with the reported literature. This may be explained by differences in gastric transit times for different types of food [31]. Future studies should evaluate postprandial brachial artery dilation at different time points to assess the effect of time. Several studies have examined FMD serially over time following meal ingestion[40,41].

While rich in cholesterol, eggs are also nutritious. Data from NHANES III reveal that egg consumption is an important nutritional contribution to the average American diet, providing a relatively inexpensive source of amino acids and essential fatty acids[9]. Eggs provide arginine, a precursor to nitric oxide, which in turn plays a central role in endothelial function[42]. Endothelial function is an arterial vasomotor response mediated predominantly by the release of nitric oxide (vasodilating), and endothelin (vasoconstricting) from the vascular endothelium[26]. This system plays an important role in the pathogenesis of atherosclerosis, cardiovascular disease, and other chronic diseases[19,43].

The relative importance of dietary cholesterol to cardiovascular risk, and the association between dietary and serum cholesterol, are both subject to ongoing debate[44-46]. The association between dietary cholesterol and coronary events and mortality is generally positive but rather weak, and derived largely from ecological and prospective cohort studies with variable follow-up[47-50]. In most of these studies, it is difficult to determine the effects of cholesterol independent of dietary fat. One large prospective cohort followed U.S. male physicians for over 20 years. The study monitored egg consumption and documented new cases of heart failure during follow-up. Results failed to find a correlation between occasional egg consumption and heart failure, although an increased risk of heart failure was related to participants who reported consuming more than one egg per day[49]. Another study found no impact of egg intake on cardiovascular risk, specifically stroke, ischemic stroke, and coronary artery disease. However, Nakamura et al demonstrated significant correlation with serum cholesterol concentrations in women consuming more than two eggs per day [51]. In a recent study by Djoussé et al, egg consumption was associated with increased risk of diabetes [52]. Djoussé et al also linked egg consumption to increased mortality and even more so in diabetics [53]. Overall, scientific studies of the relationship between egg consumption, cardiovascular disease and mortality[30,54], have thus been somewhat inconsistent.

Little, if any, epidemiological evidence exists supporting a direct link between egg consumption and cardiovascular disease or mortality risk. Previous studies have shown weak positive associations between intake of dietary cholesterol and serum cholesterol, while others failed to find any association[30]. Hu and colleagues analyzed data from two large cohorts, the Health Professional Follow-Up Study and the Nurses' Health Study, to assess the effect of egg consumption on cardiovascular events and deaths[47]. After a mean of 8 years of follow-up, no overall significant association was observed between egg consumption and risk of CHD in both males and females. Hu et al [47] also reported that the relative risk of CHD was the same whether the participants consumed less than, or more than, one egg per week.

While our study provides valuable data regarding egg ingestion in hyperlipidemic adults, it is not without limitations. The study's small sample size almost entirely derived from one community in Southwestern Connecticut may limit the generalization these of results. Furthermore, the duration of egg consumption during this study limits the ability to predict the long-term effects. Variables potentially confounding the correlation between nutrient intakes and endothelial responses included physical activity, vasoactive medication use, and genetic factors. Unmeasured or inaccurately measured dietary intake data could also have confounded results. Three-day food diaries, used to track dietary intake, did not indicate any significant unintended changes in overall dietary pattern, although changes in diet or behavior that were not captured may have impacted findings. Adjustment for potential confounders was managed through application of strict eligibility criteria, randomization, and crossover design. Also of note, the intended provocation of endothelial dysfunction by a single meal high in saturated fat failed to demonstrate a deleterious effect. Finally, endothelial function was measured only one time after treatment assignments and not monitored for a prolonged time.

## Conclusions

In light of the persistent uncertainties and lack of observational evidence regarding the effects of egg consumption on serum cholesterol and cardiac risk, the application of this methodology and technology in further studies is appropriate, and very much needed. Short of a randomized controlled trial of egg consumption and cardiovascular events, endothelial function testing offers one of the best available means to evaluate the role of egg ingestion on cardiac risk. To date, the evidence generally mitigates against an association between moderate egg consumption and increased cardiac risk. Further testing in at-risk samples, including individuals with established coronary disease, is now justified to clarify the place of eggs in a judicious and heart-healthy diet.

## Competing interests

The authors declare that they have no competing interests.

## Author contributions

The authors contributions are as follows: DLK served as the Principal Investigator and is responsible for oversight of all study related activities, data analysis and manuscript preparation. VYN was responsible for the protocol development, data analysis, interpretation, manuscript preparation, and critical review of the paper. ZF was responsible for study management, ultrasound reading, data collection, and manuscript preparation. SD contributed to manuscript preparation. AGS contributed to manuscript preparation. All Authors have read and approved the final manuscript.
